# An Observational Study Evaluating the Safety of Neoadjuvant Immunotherapy Combined With Chemotherapy in Patients Undergoing Surgery for Non‐Small Cell Lung Cancer

**DOI:** 10.1111/1759-7714.70002

**Published:** 2025-02-12

**Authors:** Xin Ye, Jinbai Miao, Hui Li, Bin Hu

**Affiliations:** ^1^ Department of Thoracic Surgery Affiliated Beijing Chaoyang Hospital of Capital Medical University Beijing China

**Keywords:** immunotherapy, neoadjuvant chemotherapy, stage III non‐small cell lung cancer

## Abstract

**Objective:**

This study was conducted to investigate the safety of neoadjuvant immunotherapy combined with chemotherapy in patients undergoing surgery for resectable stage III non‐small cell lung cancer (NSCLC).

**Methods:**

Overall, 68 surgical patients with stage III NSCLC who underwent neoadjuvant therapy at the Thoracic Surgery Department of Beijing Chaoyang Hospital from June 2019 to September 2021 were included, including 19 patients who underwent neoadjuvant chemotherapy combined with immunotherapy and 49 who underwent neoadjuvant chemotherapy alone. Both groups of patients were diagnosed with NSCLC before treatment and had resectable stage III tumors. The surgical duration, blood loss volume, average postoperative hospital length of stay, intensive care unit length of stay, and complication rate were compared between the two groups.

**Results:**

The group treated with neoadjuvant chemotherapy combined with immunotherapy demonstrated higher values than the group treated with chemotherapy alone for surgical duration, blood loss volume, and rate of conversion to thoracotomy; however, the differences were not statistically significant. The incidence of postoperative complications in the group treated with neoadjuvant immunotherapy combined with chemotherapy was significantly higher than that of the group treated with neoadjuvant chemotherapy alone (*p* = 0.02).

**Conclusion:**

Neoadjuvant immunotherapy combined with chemotherapy was safe and effective and did not increase the difficulty of surgery for NSCLC; however, it was associated with a higher incidence of complications than neoadjuvant chemotherapy alone (*p* < 0.05).

## Graphical abstract

Is the neoadjuvant immunotherapy combined with chemotherapy patient difficult to operative? In the stage III NSCLC, patients took immunotherapy were more likely to be develop complications, but on the other hand, it seems as difficult as usual chemistry therapy.

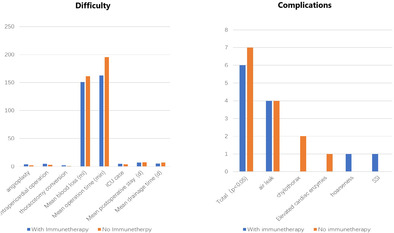

## 1 Background

Lung cancer has the highest morbidity and mortality rates of all malignant tumors worldwide, seriously threatening human life and health. Radical lobectomy combined with systematic lymph node dissection has long been the preferred treatment for lung cancer. With improvements in health awareness and the popularity of high‐resolution computed tomography, early diagnosis of lung cancer is becoming increasingly achievable. However, some patients are still not diagnosed until the advanced stages of lung cancer, and these patients miss the opportunity to undergo surgery.

Traditional chemotherapy and radiotherapy exhibit low efficacy, only increasing overall survival and progression‐free survival by 5% [1], and the major pathologic response and pathologic complete response rates to these therapies are less than 10% [2, 3]. In the past, there was no better treatment than chemotherapy for patients with lung cancer, so the prognosis of these patients was poor and the survival time was short. However, with the rise of immunotherapy, which has demonstrated extraordinary experimental and clinical results, stage III non‐small cell lung cancer (NSCLC) has entered a new treatment era. With that, the prognosis of patients with stage III NSCLC, who were previously considered inoperable, is improving. Unlike targeted therapies, immunotherapy attacks cancer cells directly, and it clears tumor cells by “waking up” the patient's immune system.

A recent study showed that neoadjuvant immunotherapy is reasonable and practical. The majority of tumor cells express immune checkpoints preoperatively, and tumor antigens contribute to the activation of tumor‐infiltrating lymphocytes during immunotherapy, leading to long‐lasting anti‐tumor effects. The systemic immune response leads to the production of long‐term immune memory and prevents tumor recurrence preoperatively. However, after tumor resection, owing to the lack of tumor cells, the systemic immune response cannot produce sustainable anti‐tumor effects [4]. A previous study [5] showed that perioperative cisplatin and docetaxel chemotherapy combined with PD‐L1 inhibition was safe. The 1‐year event‐free survival rate was high, reaching 73.3%, exceeding that of chemotherapy alone (48%), with high primary pathological response (60%) and lymph node decline rates.

This study was conducted to investigate the safety of neoadjuvant immunotherapy combined with chemotherapy in patients undergoing surgery for resectable stage III NSCLC.

## 2 Materials and Methods

### 2.1 Study Design

This study collected the data of patients with stage III NSCLC who underwent neoadjuvant therapy at the Thoracic Surgery Department of Beijing Chaoyang Hospital from June 2019 to September 2021, including those who underwent neoadjuvant chemotherapy combined with immunotherapy and those who underwent neoadjuvant chemotherapy alone. The safety of surgery was compared between the two groups in terms of surgical duration, blood loss volume, intensive care unit length of stay, volume and time of drainage, and length of postoperative hospital stay.

### 2.2 Eligibility Criteria

The inclusion criteria were (1) NSCLC confirmed by pathology; (2) preoperative tumor stage III, excluding N3 lymph node metastasis; (3) no contraindication after neoadjuvant therapy, and surgical treatment was feasible; and (4) good cardiopulmonary function. The exclusion criteria were (1) disease progression after neoadjuvant therapy or distant metastasis resulting in inoperable disease and (2) poor cardiopulmonary function that could not tolerate surgery.

### 2.3 Treatment

The treatment plan was neoadjuvant chemotherapy with a platinum‐containing dual‐agent regimen or neoadjuvant chemotherapy combined with immunotherapy using a platinum‐containing dual‐agent and immune drug regimen.

### 2.4 Ethics

The study was performed in accordance with the Declaration of Helsinki (as revised in 2013) and approved by the Ethics Committee of Beijing Chao‐yang Hospital. All patients provided written informed consent.

### 2.5 Statistical Analysis

The data are presented as the mean ± standard error. SPSS 20.0 statistical software was used for the statistical analysis. The independent‐samples *t‐*test was used to compare the data between the two groups. *p* < 0.05 was considered statistically significant.

## 3 Results

The baseline characteristics of the two groups are shown in Table 1. Surgical information is shown in Table 2, and complications are shown in Table 3.

**TABLE 1:** Baseline information of the patients.

Basic information		Chemotherapy (*n* = 49)	Chemotherapy and immune therapy (*n* = 19)	*p*
Age	≥ 65	16	9	*0.02*
	< 65	33	10	
Gender	Male	34	17	*0.05*
	Female	15	2	
Smoke index		393.3	884.2	*0.002*
Location	Central	19	11	0.16
	Peripheral	30	8	
Operation	Lobectomy	31	8	0.07
	Total pneumonectomy	7	2	
	Sleeve resection	7	7	
	Combined lobectomy	3	2	
Pathology	Squamous cell carcinoma	19	2	*0.01*
	Adenocarcinoma	30	17	

*Note:* Italic indicates significant values.

**TABLE 2:** Surgical information.

Operation information		Chemotherapy (*n* = 49)	Chemotherapy and immune therapy (*n* = 19)	*p*
Surgical method	VATS	25	6	0.15
	Open	24	13	
Angioplasty		4	2	0.76
Intrapericardial operation		5	3	0.53
Thoracotomy conversion		2	1	0.83
Mean blood loss (mL)		150.82	161.58	0.81
Mean operation time (min)		162.55	195.26	0.07
ICU case		5	4	0.31
Mean postoperative stay (d)		7.02	7.89	0.46
Mean drainage time (d)		5.51	6.79	0.26
Mean drainage volume (mL)	D1	281.12	350.53	0.28
	D2	272.86	455.00	*< 0.01*
	D3	205.74	260.53	0.06

*Note:* Italic indicates significant values.

**TABLE 3:** Complications.

Complications	Chemotherapy (*n* = 49)	Chemotherapy and immune therapy (*n* = 19)	*p*
Total	6	7	*0.02*
Air leak	4	4	0.14
Chylothorax	0	2	0.08
Elevated cardiac enzymes	0	1	0.28
Hoarseness	1	0	1
SSI	1	0	1

Overall, 68 patients were included in the study. Of these, 19 patients were included in the neoadjuvant chemotherapy combined with immunotherapy group (experimental group), including 17 males and two females. Forty‐nine patients were included in the chemotherapy alone group (control group), including 34 males and 15 females. The age range of the patients in the experimental group and control group was 51–77 years (mean 64.11 ± 6.24 years) and 36–75 years (mean 59.00 ± 8.64 years), respectively. The average smoking index in the experimental and control groups was 884.2 and 393.27, respectively. Central‐type and peripheral‐type NSCLC were observed in 11 and eight patients in the experimental group, respectively, and in 19 and 30 patients in the control group, respectively. There were two cases of adenocarcinoma and 17 cases of squamous cell carcinoma in the experimental group, while there were 30 cases of adenocarcinoma and 19 cases of squamous cell carcinoma in the control group. Lobectomy was performed in eight patients, pneumonectomy in two, sleeve resection in seven, and combined lobectomy in two. Six patients underwent thoracoscopic surgery, of which one was converted to thoracotomy, with a conversion rate of 16.67%, while 11 patients underwent open surgery. In the control group, lobectomy was performed in 31 patients, pneumonectomy in seven, sleeve lobectomy in nine, and combined lobectomy in two. There were 25 cases of thoracoscopic surgery, of which two cases were converted to thoracotomy (8%) and 24 cases underwent open surgery. In the experimental group, arterial angioplasty was performed in two patients, both of whom underwent open surgery. In the control group, arterial angioplasty was performed in four patients under open conditions. The transpericardial approach was used in three and five cases under open conditions in the experimental and control groups, respectively. The blood loss volume was 20–1000 mL (mean 161.58 ± 228.14 mL) in the experimental group and 40–600 mL (mean 150.82 ± 126.62 mL) in the control group. In the experimental group, two patients received blood transfusion during surgery and one patient received blood transfusion after surgery. In the control group, there were no cases of blood transfusion during or after surgery. The surgical duration range in the experimental group was 90–365 min (mean 195.26 ± 79.94 min), while it was 60–420 min (mean 162.55 ± 60.60 min) in the control group. After surgery, four (21.05%) and five (10.2%) patients in the experimental and control groups, respectively, were transferred to the intensive care unit. The mean length of stay in the intensive care unit was 4.25 days in the experimental group and 3.6 days in the control group. The mean postoperative hospital length of stay was 7.89 (3–15) days and the mean drainage length was 6.79 (3–15) days in the experimental group. In the control group, the mean postoperative hospital length of stay was 7.02 ± 4.61 days (range 2–25 days) and the mean postoperative drainage length was 5.51 ± 4.34 days (range 2–24 days). In the experimental group, the drainage volume range was 0–925 mL (mean 350.53 ± 58.09 mL) on the first day after surgery, 200–1250 mL (mean 455.00 ± 58.45 mL) on the second day after surgery, and 100–475 mL (mean 260.53 ± 23.36 mL) on the third day after surgery. In the control group, the drainage volume range was 0–850 mL (mean 281.12 ± 24.48 mL) on the first day after surgery, 0–650 mL (mean 272.86 ± 23.12 mL) on the second day after surgery, and 0–625 mL (mean 197.35 ± 16.83 mL) on the third day after surgery. Postoperative complications occurred in nine patients (47.37%) in the experimental group, including air leakage in six patients and chylothorax in two patients. One patient underwent a second surgical ligation of the thoracic duct, and one patient had elevated myocardial enzymes. No perioperative deaths occurred. Six patients (12.24%) had postoperative complications in the control group, including four (8.16%) with air leakage (more than 7 days), one (2.04%) with surgical site infection (SSI), and one (2.04%) with hoarseness.

In summary, our results show that neoadjuvant immunotherapy combined with chemotherapy was safe and effective, and there was no significant difference in the surgical duration, blood loss volume, length of hospital stay, or rate of conversion to thoracotomy between the group treated with neoadjuvant immunotherapy combined with chemotherapy and the group treated with neoadjuvant chemotherapy alone. However, in the group treated with neoadjuvant immunotherapy combined with chemotherapy, the complication rate was higher than that of the group treated with neoadjuvant chemotherapy alone (*p* < 0.05).

## 4 Discussion

The advent of immunotherapy has greatly improved the pathologic remission rate of patients with NSCLC. Compared with neoadjuvant single‐agent or dual‐agent immunotherapy, immunotherapy combined with chemotherapy achieves a higher major pathologic response rate [6]. The reason may be that while chemotherapy directly kills tumor cells, it also regulates the immune response.

Specifically, chemotherapy regulates the number of immune cells, kills tumor cells, and causes the release of tumor‐specific antigens (TSA) [7, 8]. The direct killing effect on tumor cells also leads to the release of tumor‐specific antigens when tumor cell apoptosis occurs. Moreover, clinical studies have shown that some chemotherapeutic drugs exert their anti‐tumor effects by affecting immune cells in the tumor microenvironment [9]. Additionally, some chemotherapeutic agents stimulate tumor cells to secrete immune‐related factors. These factors promote immune function by activating the differentiation and maturation of dendritic cells, the response of type I interferon, and the recruitment of T cells [10]. Furthermore, chemotherapy reduces the number of suppressive immune cells. For instance, gemcitabine and cisplatin specifically reduce chemotherapy‐sensitive immunosuppressive cells, such as myeloid‐derived suppressor cells and regulatory T cells, among other cell types [11, 12, 13]. In vivo, the combination of immunotherapy and chemotherapy exerts a synergistic effect through the various mechanisms described above to enhance the anti‐tumor effect. In mouse experiments, gemcitabine promoted the release of TSA and enhanced the activity of CD8^+^ T cells. Moreover, it significantly reduced the content of myeloid‐derived suppressor cells.

Chemotherapy also inhibits tumor immune escape mechanisms. Chemotherapy upregulates the co‐stimulatory factor receptor (B7‐1) on the surface of tumor cells and downregulates the inhibitory factor receptor (PD‐L1) [8]. Moreover, after immunotherapy, activated immune cells and cytokines enhance the sensitivity of tumor cells to chemotherapy, making tumor cells more sensitive to the killing effects mediated by T cells through the Fas/Fas‐1 and granzyne B pathways.

### 4.1 Surgical Safety Issues After Neoadjuvant Immunotherapy

The safety of surgery after neoadjuvant immunotherapy is of particular interest to surgeons, but there is a lack of objective evaluation indicators for this. At present, surgical safety is generally determined based on whether surgery is delayed, the duration of surgery, the blood loss volume, the surgical method (thoracoscopy, open surgery, or conversion to thoracotomy), the surgical method (lobectomy, wedge resection, pneumonectomy), and the hospitalization time, among other factors. The available evidence suggests that neoadjuvant single‐agent immunotherapy combined with chemotherapy does not lead to a large number of surgical delays. The results of the NEOSTAR study [14] showed that the median time from the last treatment to surgery was 31 days (21–87 days). Three patients (14%) in the single‐agent group and five (31%) in the dual‐agent group were waiting longer than 42 days to undergo surgery. Moreover, Bott et al. [15] reported that patients treated with neoadjuvant nivolumab did not experience surgical delay. The main reasons for the difficulty of surgery are (1) the large tumor size or invasion to important tissues and organs and (2) tissue fibrosis, increased vascular fragility, and increased difficulty of lymph node dissection [16]. In another study [17], arterial fibrosis was the most common complication in patients receiving either neoadjuvant chemotherapy or neoadjuvant chemotherapy combined with immunotherapy, with 14% of patients treated with neoadjuvant chemotherapy developing arterial fibrosis compared with 8% of patients treated with neoadjuvant combination therapy. There were no significant differences in surgical complications between the two treatment methods. The LCMC3 study also showed extensive hilar fibrosis in 20 of 90 patients (22.2%). In addition, patients who achieved a major pathologic response after neoadjuvant immunotherapy were more likely to exhibit fibrotic changes in the hilar. Bott et al.'s study included 13 patients who underwent minimally invasive thoracoscopic surgery, and seven patients were converted to thoracotomy to complete tumor resection, revealing the influence of neoadjuvant immunotherapy on surgery. Data from NEOSTAR [14] showed that 27 patients underwent thoracotomy, seven underwent thoracoscopic surgery, and three underwent robotic surgery. However, the transition thoracotomy rates were not reported. The median surgical duration was 147 (71–315) minutes, the median blood loss volume was 100 (50–1000) mL, and the median length of hospital stay was 4 (1–18) days. Although the difficulty of some types of surgery has increased, the incidence of postoperative complications has not increased significantly. In updated data from the LCMC3 study, 54% of patients underwent minimally invasive surgery, the conversion rate to thoracotomy was 15%, and only 3% of the total enrolled population experienced intraoperative bronchial or vascular complications [17]. Evidence suggests that neoadjuvant therapy induces tissue fibrosis, and its impact on surgery requires more quantitative criteria for evaluation and research.

The benefits and risks of neoadjuvant therapy coexist, with the risks including direct drug side effects, interruption of subsequent treatment, and even an increased incidence of surgical complications. Therefore, there is usually a certain interval between neoadjuvant therapy and surgery. This is because the response of the tumor after drug treatment requires a certain time window, and the toxic side effects of neoadjuvant therapy also need time to relieve before patients undergo subsequent treatment. Surgery performed at an improper time window after neoadjuvant therapy may cause extra difficulties in the surgical field.

In this study, the surgeons felt that patients treated with neoadjuvant chemotherapy combined with immunotherapy were more difficult to operate on as they frequently presented adhesions and fibrosis. That said, there was no significant difference in many of the variables compared between the groups, which may be due to the sample size being underpowered to detect significant differences.

### 4.2 Future Perspectives

In future research, there are still many issues that need to be explored with regard to neoadjuvant immunotherapy, including the treatment cycle, drug dose, use or non‐use of radiotherapy, and the operation time window, as well as the impact of immunotherapy on surgery and complication rates. Monitoring preoperative adverse reactions and postoperative adjuvant therapy and follow‐up are also important considerations. Moreover, how to exclude pseudo‐progression to avoid patients missing treatment is also a problem that requires attention. The number of patients included in the present study may have caused statistical bias, so the sample size should be expanded in future research. Moreover, neoadjuvant immunotherapy combined with chemotherapy has various possible regimens, which may have different effects on surgery, which is also a topic for evaluation.

Existing clinical data illustrate the great potential of neoadjuvant immunotherapy combined with chemotherapy for the treatment of patients with locally advanced NSCLC. Immunotherapy combined with chemotherapy is widely used to treat patients with NSCLC, but the mechanisms of its effects are still not completely clear, warranting further exploration. The design of regimens combining immunotherapy with chemotherapy has also attracted the attention of researchers. Whether simultaneous or sequential therapy is best remains to be clarified. Moreover, it remains to be clarified whether the doses of each individual therapy should be reduced or whether the regular dose should be maintained when used as part of a combination regimen. Furthermore, future research should determine what time interval after neoadjuvant therapy is associated with the lowest complication rates. For patients with severe adverse reactions after immunotherapy, whether there are biological indicators that can predict the occurrence of these adverse reactions and potentially prevent them should be determined. Another avenue of research is determining whether markers of efficacy, such as epidermal growth factor receptor‐tyrosine kinase inhibitor (EGFR‐TKI), can screen out high‐benefit groups before treatment. The data of follow‐up clinical studies should clarify the relationship between the pathological evaluation index of major pathologic response/pathologic complete response, as well as the survival indices of disease‐free survival and overall survival. Finally, the optimal adjuvant treatment mode for patients undergoing surgical resection after neoadjuvant immunotherapy remains to be explored.

## 5 Limitations

This study has some limitations that should be considered. First, the sample size was small, so the robustness of the observations is unclear. Therefore, large‐sample studies are needed to validate our findings. Second, the observational design of the study may have introduced bias. The findings may not reflect real‐world data, so further randomized controlled trials are needed. Finally, in the experimental group, patients used different types of immune therapy (PD‐1/PD‐L1), which may have influenced the results.

## 6 Conclusions

Neoadjuvant immunotherapy combined with chemotherapy was safe and effective and did not increase the difficulty of surgery in patients with NSCLC. However, it was associated with a higher incidence of complications than neoadjuvant chemotherapy alone.

## Author Contributions

Xin Ye, Jinbai Miao, and Hui Li conceptualized and designed the study. Xin Ye and Hui Li contributed to the methodological development. Xin Ye performed data collection and analysis. Xin Ye and Jinbai Miao drafted the manuscript, while Bin Hu was responsible for reviewing and editing. All authors have reviewed and approved the final version of the manuscript.

## Conflicts of Interest

The authors declare no conflicts of interest.

## Data Availability

Data can be available from the corresponding author upon reasonable request.
